# Effects of oral contraceptive use on muscle strength, muscle thickness, and fiber size and composition in young women undergoing 12 weeks of strength training: a cohort study

**DOI:** 10.1186/s12905-022-01740-y

**Published:** 2022-05-10

**Authors:** Eun-Sook Sung, Ahreum Han, Timo Hinrichs, Matthias Vorgerd, Petra Platen

**Affiliations:** 1 Department of Physical Education, Korea University, Seoul, Republic of Korea; 2 Department of Sports Medicine and Sports Nutrition, Faculty of Sport Science, Ruhr-University Bochum, Gesundheitscampus Nord, Haus 10, 44801 Bochum, Germany; 3 Division of Sports and Exercise Medicine, Department of Sport, Exercise and Health, University of Basel, Basel, Switzerland; 4 Department of Neurology, Kliniken Bergmannsheil, Ruhr-University Bochum, Bochum, Germany

**Keywords:** Menstrual cycle, Birth control pill, Resistance training, Muscle fiber distribution

## Abstract

**Background:**

It is suspected that hormonal fluctuations during menstruation may cause different responses to strength training in women who use oral contraceptives (OC) versus those who do not. However, previous studies that investigated the existence of such differences produced conflicting results. In this study, we hypothesized that OC use has no effect on muscle strength and hypertrophy among women undergoing strength training. Thus, we compared the differences in muscle strength and thickness among women who used OCs and those who did not.

**Methods:**

We investigated the influence of OC use on muscle strength (F_max_), muscle thickness (Mtk), type 1-to-type 2 muscle fiber (NO) ratio, muscle fiber thickness (MFT), and nuclear-to-fiber (N/F) ratio. Seventy-four healthy young women (including 34 who used OCs and 40 who did not) underwent 12 weeks of submaximal strength training, after which F_max_ was evaluated using a leg-press machine with a combined force and load cell, while Mtk was measured using real-time ultrasonography. Moreover, the NO ratio, MFT, and N/F ratio were evaluated using muscle needle biopsies.

**Results:**

Participants in the non-OC and OC groups experienced increases in F_max_ (+ 23.30 ± 10.82 kg and + 28.02 ± 11.50 kg respectively, *p* = 0.073), Mtk (+ 0.48 ± 0.47 cm^2^ and + 0.50 ± 0.44 cm^2^ respectively, *p* = 0.888), F_max_/Mtk (+ 2.78 ± 1.93 kg/cm^2^ and + 3.32 ± 2.37 kg/cm^2^ respectively, *p* = 0.285), NO ratio (type 2 fibers: + 1.86 ± 6.49% and − 4.17 ± 9.48% respectively, *p* = 0.169), MFT (type 2 fibers: + 7.15 ± 7.50 µm and + 4.07 ± 9.30 µm respectively, *p* = 0.435), and N/F ratio (+ 0.61 ± 1.02 and + 0.15 ± 0.97 respectively, *p* = 0.866) after training. There were no significant differences between the non-OC and OC groups in any of these parameters (*p* > 0.05*)*.

**Conclusions:**

The effects of 12 weeks of strength training on F_max_, muscle thickness, muscle fiber size, and composition were similar in young women irrespective of their OC use.

## Background

Second-generation monophasic oral contraceptives (OCs) are increasingly being used by women worldwide for controlling their menstrual cycles, managing premenstrual symptoms and dysmenorrhea, reducing menstrual blood loss, and regulating the timing of menstruation [1–4]. The increased participation of women in sports has sparked additional interest in understanding their physiological responses to exercise and muscle exertion [5, 6]. Studies on the relationship between the menstrual cycle and sports performance in women based on whether they use OCs often focus on the hormones involved in menstruation [7, 8].

The most commonly used second-generation monophasic OCs comprise ethinylestradiol and progestin in fixed doses and are taken continuously for 21 days (the consumption phase), during which the production of endogenous estradiol and progesterone is continuously suppressed, followed by a 7-day break (the withdrawal phase) [3]. Ethinylestradiol is highly associated with venous thromboembolism; hence, its dose in OCs has been lowered to 30–35 µg to reduce the risk of such adverse effects. Moreover, it was recently reported that a dose of 20 µg can be used without any apparent loss of contraceptive efficacy [9].

In contrast, endogenous female sex hormones affect various body functions including exercise performance [10]. Follicular phase-based strength training has been found to produce a more pronounced effect on performance than luteal phase-based training, which is likely due to the specific hormonal milieu during each phase of the menstrual cycle [11]. Sex hormones such as estrogen and progesterone are steroids with similar molecular structures, and various factors can influence their secretion during exercise [12, 13].

Studies of the effect of OCs on women athletes are ongoing [14]. Because sex hormones play a significant role in exercise adaptation, OC-induced changes in their blood concentrations may reduce the magnitude of such adaptation, thereby impairing strength performance [15]. Indeed, certain studies found that OC use reduces maximal exercise capacity [16, 17] and impedes peak adaptation to training [18], while others failed to detect significant effects of OC use on muscle strength [7, 19, 20]. OC use may limit the potential anabolic effects of estrogen on muscle strength owing to decreased fluctuations in hormone levels during the menstrual cycle [21].

Although previous studies investigated the effect of OC use on exercise adaptation, its impact on exercise performance is poorly understood. According to Nichols et al., OC use had no significant effect on strength development compared to no OC use [22]; however, Ruzic et al. found that OC use was associated with significantly greater gains in muscle strength and lean mass [23]. Given these inconsistent data, the roles of hormonal OCs in exercise performance remain unclear; this lack of understanding is often noted in publications about menstrual cycle and strength [10]. In this study, we hypothesized that OC use would have no effect on muscle strength and hypertrophy among women undergoing strength training. To that end, we compared the differences in muscle strength and thickness among women who used OCs and those who did not; additionally, we compared muscle fiber composition, muscle fiber thickness (MFT), and muscle nucleus-to-fiber (N/F) ratio between these 2 groups after the participants underwent 12 weeks of strength training.

## Methods

### Participants

Seventy-four young and healthy female students from Ruhr-University Bochum participated in this study (Table 1). The participants were not conditioned to submaximal resistance training but were otherwise active (i.e., they performed less than 2 h of exercise such as yoga or aerobics weekly during the preceding 6 months); some had been using a bicycle for transportation for at least 1 year. They had regular menstrual cycles for at least the preceding year and had no history of endocrine disorders.

**Table 1:** Participants’ characteristics

Group	non-OC (n = 40)	OC (n = 34)	*p*-value
Age (years)	25.00 ± 4.56	22.39 ± 2.30	0.016
Height (m)	1.64 ± 0.05	1.67 ± 0.06	0.340
Weight (kg)	61.14 ± 8.36	63.89 ± 9.44	0.620
BMI (kg/cm^2^)	22.69 ± 1.73	22.91 ± 2.68	0.993

Non-OC, women not taking oral contraceptives; OC, women taking oral contraceptives; BMI, body mass index. Values are presented as means ± standard deviations

The participants were categorized into 2 groups: non-OC (no OC use or hormonal treatment in the past year) and OC (use of OC for at least 1 year immediately preceding the study). Those in the OC group used second-generation monophasic combined OCs with ethinylestradiol doses between 20 and 30 µg; 3 used Anastrella 28 (Orifarm Generics AB, Stockholm, Sweden), 10 used Cilest (Janssen Pharmaceutica, Beerse, Belgium), 8 used Femicept (CampusPharma, Göteborg, Sweden), 9 used Loette (Pfizer, West Ryde, Australia), and 4 used Microstad (STADA Nordic, Herlev, Denmark). Each participant provided written informed consent prior to enrollment in the study after being informed of the purpose and procedures involved as well as the associated risks. This study has been verified and approved by the ethics committee of Ruhr-University Bochum, Germany (IIA1-070118/07), and written informed consent has been obtained from all the included participants. All the study procedures were carried out in accordance with the principles of the Declaration of Helsinki, 1964 and its later amendments.

### Monitoring of the menstrual cycle phase in the non-OC group

Changes in body temperature were used to identify the phases of the menstrual cycle, based on which the training and testing schedule was formulated. Participants were requested to use an oral electronic thermometer to measure their temperature for 1 min daily between 8:00 and 8:30 am before rising from bed, for 5 months [24]. The readings for the first 2 months were used to check the regularity of the menstrual cycle for participants in the non-OC group. Ovulation was considered to have occurred when an increase of at least 0.3℃ in oral temperature was noted [25–27]. When the regularity of the menstrual cycle using this method over 2 consecutive months was confirmed, strength training began on the first day of menstrual bleeding in the third month. Women in the non-OC group continued to have regular menstrual cycles (28.3 ± 1.2 days); there was no significant difference in the cycles between the two groups of women.

### Experimental design

The entire study spanned 5 menstrual cycles, which was equivalent to approximately 140 days considering that a single menstrual cycle lasted approximately 28 days. The following parameters were measured in the non-OC group. During the first menstrual cycle, only the body temperature was monitored. During the second cycle, body temperature was monitored and was noted before measuring the strength of maximum isometric knee extension (F_max_) and the sum of the thicknesses of the rectus femoris, vastus intermedius, and vastus lateralis muscles (Mtk); moreover, a biopsy of the vastus lateralis muscle was performed on participants who consented to it. During the third and fourth cycles, body temperature was monitored while the subjects underwent strength training. Finally, during the fifth cycle, the subjects underwent temperature monitoring, strength training, and final measurements of the F_max_ and Mtk; moreover, another vastus lateralis muscle sample was obtained. We did not control the participants’ diets during the intervention period; however, they were instructed not to change their eating habits, and none reported any such change upon inquiry.

### Strength training program

The participants completed 12 weeks (i.e., 3 consecutive menstrual cycles) of submaximal strength training. The initial training value was set to 85% of the maximal isometric strength test, and participants performed 3 sets of 8–12 repetitions on a leg-press machine (Selection Pro Leg press, Technogym®, Cesena FC, Italy) with 2 min of resting intervals between sets until exhaustion [28]. If any participant was able to perform more than 12 repetitions during a set, we increased the resistance by 10 kg (i.e., 85–95% of their maximal strength) to reduce the number of repetitions to 12 or less.

Supervised training was performed 3 times a week (Mondays, Wednesdays, and Fridays) at our training laboratory between 1 p.m. and 6 p.m. using the leg-press machine and once a week (Saturdays) at home using the participant’s own body weight (squats). For the latter exercise, participants performed 3 sets of 15–20 leg squats with 3–5 min of recovery between sets.

### F_max_ measurement

The F_max_ was measured using a leg-press machine (Medizinische Sequenzgeräte, Compass, Germany) with a combined force and load cell (GSV-2ASD, ME-Messsysteme GmbH, Hennigsdorf, Germany). The laboratory temperature was 20℃ with 50% humidity. Each participant’s F_max_ was measured on the 25^th^ day of their menstrual cycle. Before testing, the test method was explained to the participants and 2 practice runs were performed, after which the participants underwent a 10-min warm-up on a low-resistance bicycle ergometer. They were made familiar with the leg-press task and testing position (i.e., knees were flexed at a 90° angle). Each measurement was repeated 3 times, with rest pauses of 30 s between trials. The highest value among the 3 measurements was selected for data analysis. Moreover, an analysis of reliability was performed for the isometric measurement; the intra-class correlation coefficient was 0.998, which indicated that the system had high internal consistency and thus high reliability.

### Mtk measurement

The thicknesses of the rectus femoris, vastus intermedius, and vastus lateralis in each leg were measured using real-time ultrasonography, which was previously shown to be reliable [29]. We analyzed the distances between the outer and inner muscle fasciae using a Vivid I CE 0344 ultrasonography device (GE Medical System, Solingen, Germany) with a parallel scanner (8 L-RS, 4.0–13.3 MHz) that provided a sound wave penetration depth of 10 cm, thereby enabling deep muscle visualization. Measurement of the cross-sectional areas of the most superficial part of the muscle, which is a previously validated method when using real-time ultrasonography [30] was performed before training; the participants were first made to lie down for at least 30 min to avoid muscle tension [31]. The transducer was held at an angle of 90° to the muscle border to ensure a clear image; the images were then captured on the screen, and muscle thicknesses were measured. Ultrasonography images were obtained at a point halfway between the anterior superior iliac spine and the upper margin of the patella with the participants in the supine position; moreover, the position of the transducer was recorded for each muscle to enable precise replication during subsequent measurement. Three measurements were obtained for each muscle, and the sums of the mean thicknesses of the rectus femoris and vastus lateralis muscles were calculated for each side. Reliability analysis revealed an intra-class correlation coefficient of 0.997, indicating high reliability.

### Analysis of muscle fiber composition

Fourteen and 12 subjects in the non-OC and OC groups, respectively, consented to undergo muscle needle biopsy before and after the 12-week training program. Under local anesthesia, percutaneous muscle biopsy samples (70–300 mg) were obtained from the vastus lateralis muscle as previously described [32]. Immediately after the sample was obtained, the tissue was removed from the needle, cross-sectionally mounted on a slide in Tissue-TEK optimum cutting temperature solution (Sakura Finetek Europe B.V., Zoeterwoude, the Netherlands), frozen in isopentane, and placed into an aluminum container to be frozen in liquid nitrogen before being stored at − 80 °C for subsequent analysis. Thin sections of 10 μm each were cut in a cryostat at − 20 °C and mounted on cover glasses for staining.

**Fig. 1:**
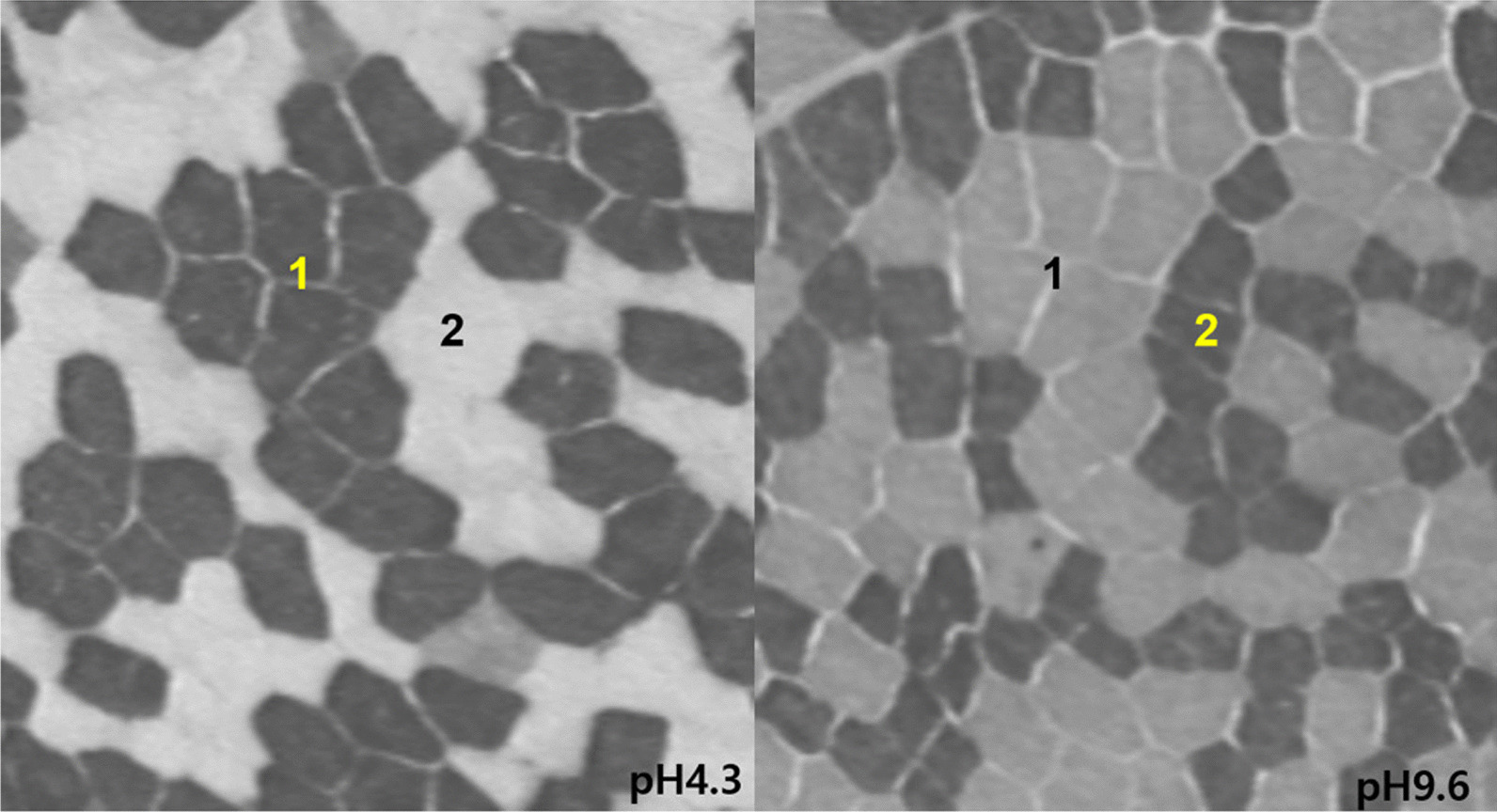
Schematic representation of muscle fibers after ATPase staining at pH 4.3 and pH 9.6

We performed histochemical analysis using adenosine-triphosphatase staining with preincubation at pH 4.3 and pH 9.6 to determine the type 1-to-type 2 muscle fiber (NO) ratio as a measure of muscle fiber type conversion [33] (Fig. 1). The different muscle fiber types were counted, and the MFTs were measured on the photographs. Muscle fiber composition was determined by identifying an average of 288 fibers from each sample and calculating the proportion of each type. To measure the MFT, an average of 62 fibers of each type (range, 20–119) were selected and analyzed using Cell-D life science documentation software (Olympus Life and Material Science Europe GmbH, Hamburg, Germany). Muscle cell nuclei were stained with hematoxylin and eosin to determine the N/F ratio [34]. Each sample was measured twice; the mean values were subjected to statistical analyses; the nuclei of all muscle cells from which the MFTs were obtained were enumerated.

### Statistical analysis

The sample size was calculated using a post hoc analysis with a power of 0.92 and a significance level (α) of 0.05 based on the effect size of 0.8, as well as previously published means and standard deviations of differences between the OC and non-OC groups, using the G*Power software [35]. Statistical analyses were performed using SPSS version 27.0 (IBM Corp., Armonk, NY, USA). Data are presented as mean values with their standard deviations. Normality of the distributions was assessed using the Kolmogorov–Smirnov test, while 1-way analysis of variance was used to analyze differences in the outcome variables between pre- and post-test performances. Non-parametric t-tests were used to analyze resistance training effects on the outcome variables among women in the OC and non-OC groups. Additionally, Tukey post hoc testing was performed for variables that showed significant differences between the non-OC and OC groups pre- and post-training (*p* < 0.05).

## Results

The F_max_, Mtk, and F_max_/Mtk significantly increased after training in both the non-OC group (F_max_, 69.31 to 92.61 [Δ + 23.30] kg; Mtk, 6.13 to 6.61 [Δ + 0.48] cm^2^; and F_max_/Mtk, 11.52 to 14.30 [Δ + 2.78] kg/cm^2^) and the OC group (F_max_, 86.54 to 114.56 [Δ + 28.02] kg; Mtk, 5.98 to 6.48 [Δ + 0.50] cm^2^; and F_max_/Mtk, 14.46 to 17.78 [Δ + 3.32] kg/cm^2^). The F_max_, Mtk, and F_max_/Mtk values within each group significantly increased after 12 weeks of strength training compared to those at baseline (*p* = 0.000 for all); however, interaction analysis revealed no significant differences in the changes in these parameters between patients in the non-OC versus OC groups (F_max_ = 0.073, Mtk = 0.894, and F_max_/Mtk = 0.283) (Table 2).

**Table 2:** Macroscopic muscle parameters before and after 12 weeks of submaximal strength training in young women

Variables	Testing	non-OC (n = 40)	OC (n = 34)	non-OC versus OC *p*	G × T
F_max_ (kg)	Pre	69.31 ± 14.08	86.54 ± 21.53	0.000	0.073
	Post	92.61 ± 16.17	114.56 ± 24.86	0.000	
	*p*	0.000	0.000		
	Δ	+ 23.30 ± 10.82	+ 28.02 ± 11.50	0.073	
Mtk (cm^2^)	Pre	6.13 ± 1.08	5.98 ± 0.57*	0.483	0.894
	Post	6.61 ± 1.16	6.48 ± 0.77	0.575	
	*p*	0.000	0.000		
	Δ	+ 0.48 ± 0.47	+ 0.50 ± 0.44	0.888	
F_max_ (kg)/Mtk (cm^2^)	Pre	11.52 ± 2.63	14.46 ± 3.28	0.000	0.283
	Post	14.30 ± 3.07	17.78 ± 3.55	0.000	
	*p*	0.000	0.000		
	Δ	+ 2.78 ± 1.93	+ 3.32 ± 2.37	0.285	

*OC_pre_ versus non-OC_post_, *p* ≤ 0.05. Non-OC, women not receiving oral contraceptives; OC, women receiving oral contraceptives; G × T, group × time interaction; Pre, pre-training; Post, post-training; F_max_, maximum isometric force; Mtk, sum of the thicknesses of the rectus femoris, vastus intermedius, and vastus lateralis muscles; F_max_/Mtk, maximum isometric force divided by muscle thickness

**Fig. 2:**
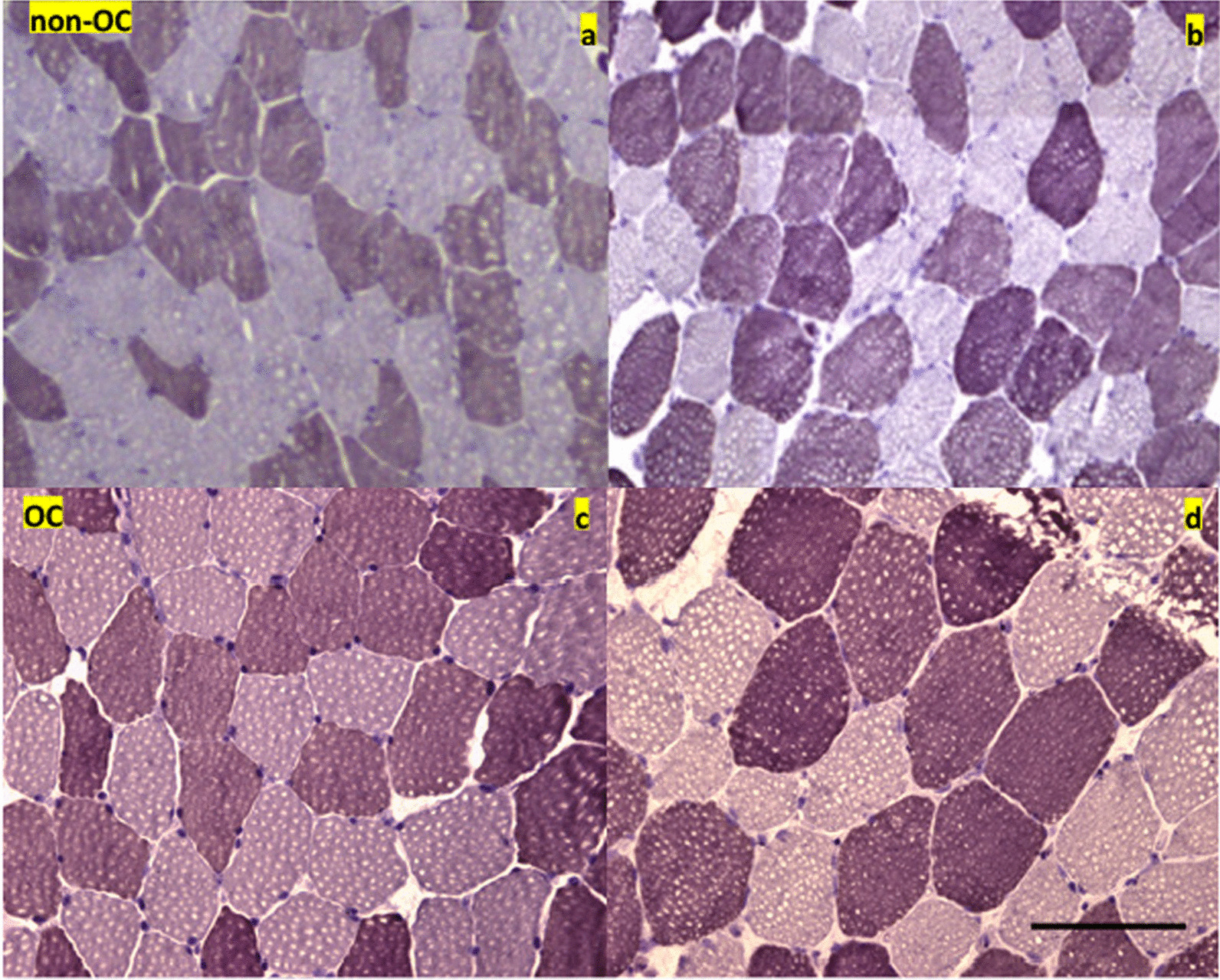
Increases in the proportions and thicknesses of type 2 muscle fibers. The photographs (× 10 magnification) compare young women in the non-OC (**a**: pre-training, **b**: post-training) and OC (**c**: pre-training, **d**: post-training) groups before and after 12 weeks of strength training. Scale bar: 100 μm. OC, oral contraceptive

**Fig. 3:**
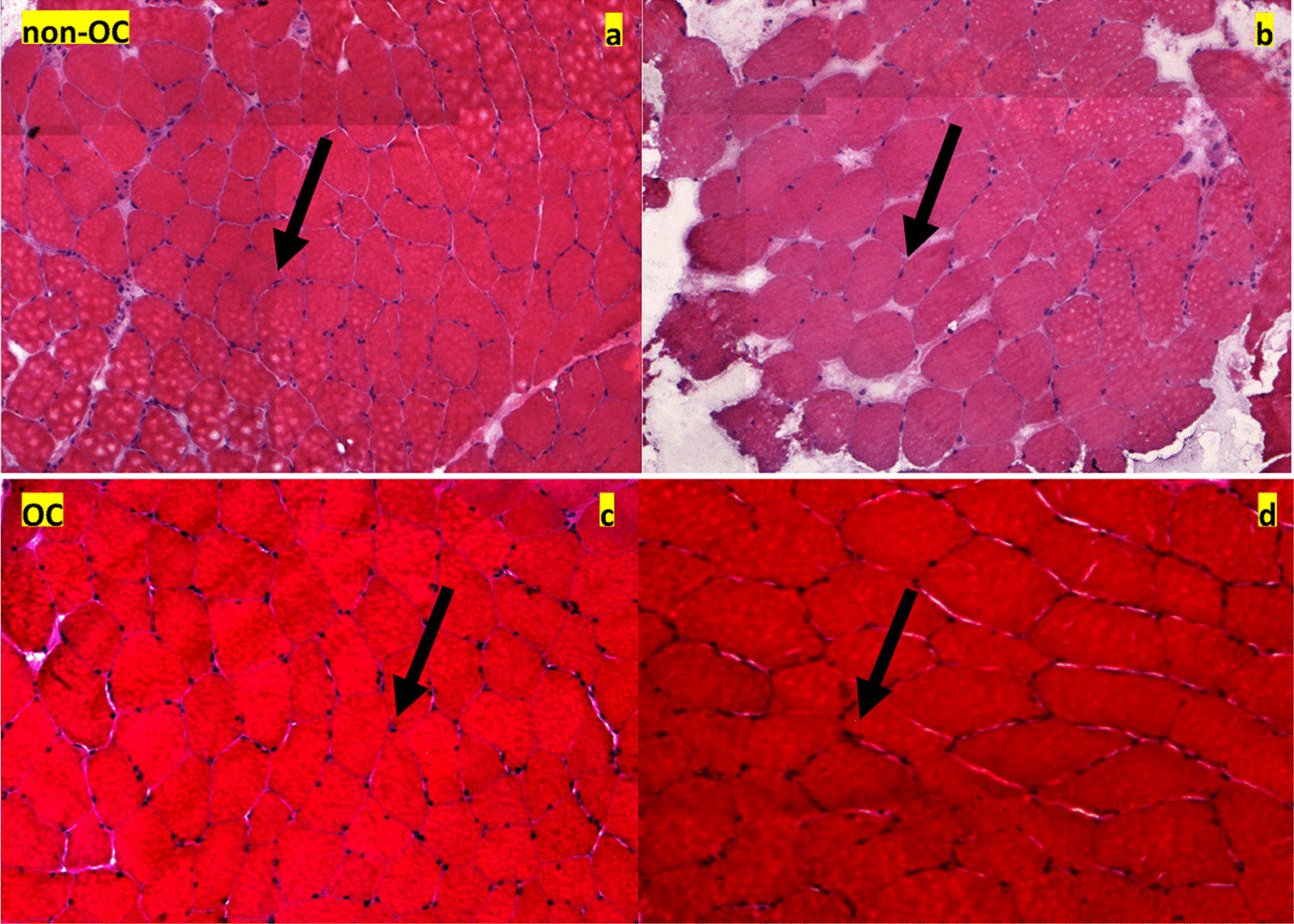
Increases in the numbers of muscle cell nuclei after 12 weeks of strength training. The panels compare women in the non-OC (**a**: pre-training, **b**: post-training) and OC (**c**: pre-training, **d**: post-training) groups. OC, oral contraceptive

The NO ratios and MFTs of type 2 fibers, as well as the N/F ratio, increased after training in the non-OC group (NO, 59.19%; MFT, 53.39 μm; and N/F ratio, 3.65) and OC group (NO ratio, 60.05%; MFT, 55.90 μm; and N/F ratio, 3.35). However, only the MFT of type 2 fibers (*p* = 0.003) and N/F ratio (*p* = 0.044) increased significantly after training in the non-OC group. When comparing between the non-OC and OC groups after 12 weeks of strength training, there were no significant differences in NO ratio, MFT, and N/F ratio (all *p* > 0.05). The analysis of interaction also revealed no significant differences in these parameters between the 2 groups (Table 3). Representative images showing the increases in type 2 fiber NO ratios and MFTs after training in members of the non-OC and OC groups are shown in Fig. 2. Moreover, representative images showing the increases in muscle cell nuclei after training are shown in Fig. 3.

**Table 3:** Microscopic muscle parameters in young women before and after 12 weeks of submaximal strength training

		non-OC (n = 14)		OC (n = 12)		Non-OC versus OC		G × T
Variables	Testing	Type 1	Type 2	Type 1	Type 2	^a^*p*	^b^*p*	
NO (%)	Pre	42.67 ± 12.52	57.33 ± 12.52	44.12 ± 15.00	55.88 ± 15.00	0.729	0.729	^1^0.840
	Post	40.81 ± 12.61	59.19 ± 12.61	35.95 ± 13.37	60.05 ± 13.37	0.762	0.747	^2^0.840
	*P*	0.305	0.283	0.156	0.156			
	Δ	− 1.86 ± 6.49	+ 1.86 ± 6.49	− 4.17 ± 9.48	− 4.17 ± 9.48	0.158	0.169	
MFT (μm)	Pre	53.43 ± 6.51	46.24 ± 7.67	53.45 ± 6.33	51.83 ± 6.79	0.771	0.784	^1^0.432
	Post	56.83 ± 6.51	53.39 ± 6.63	54.29 ± 5.95	55.90 ± 8.87	0.563	0.345	^2^0.432
	*P*	0.100	0.003	0.723	0.158			
	Δ	+ 3.41 ± 7.19	+ 7.15 ± 7.50	+ 0.84 ± 800	+ 4.07 ± 9.30	0.876	0.435	
N/F ratio	Pre	3.04 ± 0.63		3.20 ± 0.65		0.912		0.258
	Post	3.65 ± 1.02		3.35 ± 0.77		0.400		
	*P*	0.044		0.597				
	Δ	+ 0.61 ± 1.02		+ 0.15 ± 0.97		0.866		

^a^Type 1 fibers, ^b^type 2 fibers. Non-OC, women not receiving oral contraceptives; OC, women receiving oral contraceptives; G × T, group × time interaction; Pre, pre-training; Post, post-training; NO, muscle fiber ratio; MFT, muscle fiber thickness; N/F ratio, nucleus/fiber ratio

## Discussion

This study investigated Effects of oral contraceptive use on muscle strength, muscle thickness, and fiber size and composition in young women undergoing 12 weeks of strength training. We found that no OC use and OC use had similar effects on skeletal muscle adaptation to strength training in women. There are a number of studies on the influence of OC on adaptation to strength training, but their findings are conflicting [15, 36–38]. We performed a well-planned and controlled study on the effects of monophasic second-generation OCs on macroscopic and microscopic muscle parameters following strength training; these factors are important for young women who regularly engage in strength training regardless of their menstrual cycle phase.

Endogenous estrogen and progesterone levels influence substrate metabolism during exercise, as well as muscle strengthening and hypertrophy [22, 39–43]. In women of reproductive age, the levels of these endogenous sex hormones fluctuate throughout the menstrual cycle [44]; they not only influence the reproductive system but also have other physiological functions [11]. Estrogen, which reaches high levels in the follicular phase and is assumed to act as an anabolic agent, plays a role in lipid metabolism by increasing the maximal activity of key fat oxidation enzymes in skeletal muscles [45], stimulates AMP-activated protein kinase in skeletal muscles [46], and enhances growth hormone levels [47]. Conversely, progesterone, which reaches a high concentration in the luteal phase, appears to inhibit the lipolytic effect of endogenous estradiol and has catabolic effects on muscles [48, 49]. Thus, it is reasonable to assume that hormone fluctuations during the menstrual cycle among women who do not take OCs can influence the effectiveness of muscle training.

According to a previous study, the increase in the lean mass of OC users was lower than that of non-OC users, which was attributable to OC-induced changes in the androgenicity of progesterone or in anabolic/catabolic hormone levels [36]. However, we found that, while the F_max_, Mtk, and F_max_/Mtk increased significantly after training in both the OC and non-OC groups, the absolute increases in these parameters after training were not significantly different between the 2 groups. These findings indicate that OC use does not affect maximal isometric muscle strength, which is consistent with the findings of Nichols et al. that OC use does not negatively affect muscle strength gains after a 12-week free-weight strength training program because it prevents fluctuations in other anabolic hormones during the menstrual cycle [50]. Romance et al. [51] analyzed the influence of OC use on strength after 8 weeks of resistance training and found no differences in muscle strength gains between their non-OC and OC groups. Furthermore, Wikstrom-Frisen et al. found no significant differences in strength, squat jump ability, or isokinetic peak torque between women who used OCs and those who did not [52], while Nichols et al. found no differences in isokinetic torque and maximum strength gain between female student athletes who used OCs and those who did not [22]. According to Oxfeldt et al., 10 weeks of resistance training increased ‘muscle regulatory factor 4’ expression levels as well as satellite cell numbers in women who used OCs compared to nonusers [53], and Dalgaard et al. reported that the use of third-generation OCs was associated with an increase in muscle mass [54]. These data indicate that OC use does not negatively influence strength and appears not to interrupt the anabolic response of muscles to resistance exercise [15, 19, 55]. However, most of the aforementioned studies did not evaluate the microscopic parameters of muscle cells.

Based on previously published results and recommendations [11], we analyzed the NO ratios, MFTs, and N/F ratios of type 1 and 2 fibers after 12 weeks of strength training. We found that the F_max_, F_max_/Mtk, NO ratio, MFT, and N/F ratio of type 2 muscles increased in each of the non-OC and OC groups after 12 weeks of strength training, with no significant differences in these gains between the 2 groups.

Although our results do not show that OC use has a direct effect on muscle strength and hypertrophy in women, other benefits of OC use may exist. According to Hansen et al. [56], postmenopausal women who performed exercise while on estrogen supplementation therapy had a higher muscle fiber protein fractional synthesis rate than exercising counterparts who were not on such therapy; this was associated with enhanced synthesis rates of myofibrillar proteins. Dalgaard et al. found that using third-generation OCs can have a positive synergistic effect on the response to strength training at the myocellular level; combined with regular exercise, this could improve skeletal muscle signal transduction via the activation of estrogen receptors [54]. They speculated that, while endogenous estrogen levels fluctuate during the menstrual cycle, OC use causes a steady level of estrogen to be maintained; this leads to continuous stimulation of the intramuscular estrogen receptors during the first 21 days of the OC cycle, thereby increasing the anabolic effect. These data suggest that the amount of estrogen in women on OCs may not negatively influence adaptation during resistance training but may instead positively affect satellite cells and increase anabolic stimuli [57]. This could explain why third-generation OCs have no negative influence on muscle strength; instead, women using these OCs show similar improvement in muscle strength as do nonusers. We observed increases in the composition, thickness, and N/F ratio of type 2 muscle fibers in both our (second-generation) OC and non-OC groups even though the differences in these gains were not statistically significant. While there were also no significant differences in microscopic and macroscopic parameters (i.e., absolute increase in muscle strength) between the groups, we observed a positive increase in strength after the intervention (training); these findings could be of great importance to women who are hesitant to use second-generation OCs for fear of their negative effects on strength performance. However, OC use inhibits the natural production of anabolic hormones such as estrogen, and additional research is needed to investigate the effects of long-term OC use on strength.

This study had several limitations. First, although nutrition plays an important role in muscle hypertrophy, we could not investigate its effect because the nutrient intake of the participants could not be assessed. Second, because muscle biopsy was only performed in participants who consented to it, certain analyses were performed on a smaller number of participants than others. Third, given that a previous study suggested that it is necessary to measure an average of 150 muscle fibers to reduce variability [58], our lower average of 62 fibers used to determine the MFT (which was a consequence of freezing-induced damage in some muscle cells) may have been inadequate. Fourth, only 1 exercise (leg-press) was performed in this study; different results may have been obtained if a variety of exercises had been included in the training program.

## Conclusion

Skeletal muscle adaptation to exercise was similar in women who were on OCs and those who were not, and women in both groups showed similar strength trainability. Therefore, our data indicate that young women can increase muscle strength through conventional strength training irrespective of OC use.

## Data Availability

The datasets used and/or analyzed in this study are available from the first author on reasonable request.

## Abbreviations

OC: Oral contraceptives
F_max_: Strength of maximum isometric knee extension
Mtk: Sum of the thicknesses of the rectus femoris, vastus intermedius, and vastus lateralis muscles
MFT: Muscle fiber thickness
N/F ratio: Nucleus-to-fiber ratio
NO ratio: Muscle fiber ratio
